# A call to action: strengthening the capacity for data capture and computational modelling of HIV integrated care in low‐ and middle‐income countries

**DOI:** 10.1002/jia2.25475

**Published:** 2020-06-19

**Authors:** Linda E Kupfer, Blythe Beecroft, Cecile Viboud, Xujing Wang, Pim Brouwers

**Affiliations:** ^1^ Fogarty International Center US National Institutes of Health Bethesda MD USA; ^2^ National Institute of Diabetes and Digestive and Kidney Diseases US National Institutes of Health Bethesda MD USA; ^3^ National Institute of Mental Health US National Institutes of Health Bethesda MD USA

**Keywords:** HIV, NCD, integrated care, LMICs, epidemiological and clinical data, computational modelling, capacity building

Remarkable advances in HIV prevention, care and treatment, made through the discovery of antiretroviral drugs, have enabled people living with HIV (PLHIV) to live longer lives [1]. Yet, as they age, PLHIV experience co‐morbidities that require medical care, especially for non‐communicable diseases (NCD) of ageing such as cardiovascular disease, diabetes, cancer and depression and anxiety disorders [2, 3]. However, whether and how NCD treatment for PLHIV is delivered is dependent on the existing health systems and policies in the country in which a person resides.

Many low‐ and middle‐income countries (LMICs) have weak health systems with little or no NCD care or treatment available to all persons including PLHIV [4]. However, due to significant global investments to curb the HIV epidemic, HIV care in many LMICs, is delivered through clinics or other platforms that have well‐trained health personnel to provide care, electronic medical records with modules to record the chronic treatment HIV requires, HIV specific educational programmes as well as other infrastructure to diagnose and treat HIV. While many of these HIV clinics currently focus only on HIV care and perhaps co‐infections such as TB, resources could be spent to also deliver NCD care at these clinics. This type of “integrated care” would include the ability to diagnose, treat and care for comorbid NCDs in PLHIV. The term “integrated care” in this article refers to providing care for NCDs alongside HIV care at the same facility.

For integrated care to be implemented, health care personnel need to be trained and the infrastructure at the existing HIV clinics needs to be enhanced to enable diagnosis, treatment and care for NCDs in PLHIV. The NCDs covered in these clinics might be different in each country as well as in different regions of the country, depending on the burden of the specific NCDs in PLHIV. To enable integrated care, ideally each country would be able to determine its own priority NCDs for PLHIV.

The use of mathematical modelling to determine both the burden of NCDs in PLHIV in specific LMICs and the best way to deliver NCD care to PLHIV in that country, was suggested in a research agenda [5] developed through a multi‐year, multi‐agency, multi‐LMIC project led by the US National Institutes of Health (NIH) Fogarty International Center (FIC) [6]. The project “Research to Guide Practice: Enhancing HIV/AIDS Platforms to Address Non‐Communicable Diseases in Sub‐Saharan Africa” (HIV/NCD Project) investigated the current landscape of integrated HIV/NCD care for PLHIV in sub‐Saharan Africa [4] and developed a research agenda for integrated HIV/NCD care with both LMIC and US stakeholders such as ministers of health, implementers of health care and researchers. The research agenda prioritized the development of computational models to address areas such as: the cost and cost‐effectiveness of integrated HIV/NCD care; the impact of integrated care on different diseases and various at‐risk populations; the optimal approach to management of NCDs among PLHIV and how HIV and NCD care could be integrated along their care cascades – for example, how could they screen patients for hypertension and/or diabetes at the same time as HIV screening [7, 8].

Computational modelling offers useful in‐silico tools to address the pivotal areas reflected in the HIV/NCD Project research agenda. However, it is challenging for LMICs to develop computational models using local data and local capacity. Regarding local data, observational and experimental NCD data on PLHIV are seriously lacking in most LMICs where the existing HIV care systems do not collect or record NCD data. Collection of both HIV and NCD data needs to be implemented at the level of health records and national surveys. Infrastructure to systematically collect, manage, link and share NCD clinical data needs to be developed and enhanced. This effort is relevant to the upcoming NIH Common Fund programme, “Harnessing Data Science for Health Discovery and Innovation in Africa” [9]. Improved data collection and sharing should follow modern data science best practice guidelines that arise from community consensus, such as the NIH guidelines [10] and the Findability, Accessibility, Interoperability and Reusability principles [11]. Protection of human data safety is important and applies to data collection, data handling, and development and application of modelling tools. Increased availability of datasets from LMICs will empower modellers to comprehensively investigate integrated care addressing locally relevant questions and using local information.

Regarding local capacity, there is a lack of mathematical modelling training in‐country, with very few experts trained in mathematical modelling and even fewer trained in mathematical modelling of HIV/NCD integrated care. It is therefore important to develop mathematical modelling training programmes to train local researchers, who understand the local context, to model HIV and NCD integrated care. By training modellers in‐country, the hope is that the modellers will remain in‐country and will help mentor future generations. Below we discuss two types of Fogarty supported mathematical modelling training programmes, one focused on infectious disease modelling and one on HIV/NCD integrated care modelling, that we hope can inform future investments in HIV/NCD modelling training programmes in LMICs.

To begin to address the gap in modelling data and capacity for integrated care in LMICs three HIV/NCD mathematical modelling research grants were awarded by Fogarty under the HIV/NCD Project as part of a peer‐reviewed competition [12]. Each of the grants had a linked capacity building component and the three grants together provided computational modelling training to six persons from Kenya and Uganda and allowed staff from local ministries to spend time with modelling experts from the US and Europe. To analyse the research capacity portion of the grants, we used WHO/ESSENCE “Seven principles for strengthening research capacity in LMICs” as a framework [13] to design a survey and follow‐up interviews with modelling grantees to identify facilitators and barriers to building the capacity to construct computational models specifically applied to integrated HIV/NCD care in LMICs. The results of the surveys and interviews are detailed in Table 1 below. Several important facilitating factors were identified for building modelling capacity, for example, having established long‐term partnerships with institutions and researchers in the country; holding high‐income country (HIC)‐led short‐term training at LMIC academic institutions to capacitate the institutions and engage their support for this field; and having the HIC initially provide mentors until mentors in the LMIC can be established. Important barriers identified were the lack of trust for computational modelling among policy makers in‐country; the lack of long‐term institutional investment in mathematical modelling in the country, particularly as it may not be a priority scientific area for the country; unreliable internet; and a lack of recognition in both HIC and LMIC tenure track positions at academic institutions that scientific capacity building is important.

**Table 1:** Facilitators and barriers to computational modelling capacity building

Principles for sustainable capacity building^a^	Facilitators^b^	Barriers^b^
Network, collaborate, communicate and share experiences	New platforms developed for data collection and collaboration	1) Lack of infrastructure and funding; 2) Lack of institutional capacity in‐country; 3) unreliable communications networks, especially internet communications 4) lack of modelling conferences in LMICs
Understand the local context and evaluate existing research capacity	1) Established long‐term partnerships and relationships with in‐country researchers; 2) ability to conduct systematic reviews	1) No existing partnerships or relationships; 2) no experience working in LMICs or in conducting reviews
Ensure local ownership and active support	1) Having in‐country partners (MOH/academic take a lead role in project; 2) leverage previous ties and relationships	1) Distrust and/or lack of understanding of modelling; 2) distrust of sharing of data outside of country; 3) high turnover in local institutions; 4) huge workloads and competing priorities of in‐country partners
Build‐in monitoring, evaluation and learning from the start	Other successful programmes using monitoring and evaluations and capacity building, such as HIV programmes, enable more support for this type of activity	Other more pressing priorities mean that these areas are often undervalued
Establish robust research governance and support structures and promote effective leadership	1) Funding for long‐term institutional capacity building; 2) HIC mentors for leadership; 3) leadership training; 4) fellowship training at academic institutions	Lack of 1) long‐term institutional investment; 2) in‐country mentors; 3) licenses required for models; 4) locally relevant data; 5) recognition at the university level (HIC & LMIC) that capacity building is important thus no career credit to researchers who conduct training/capacity development
Embed strong support, supervision and mentorship structures	1) Initially some of the support and mentorship may have to come from the HIC partners; 2) short courses at institutions in‐country are important to generate support	Lack of knowledge about modelling leads to lack of support in LMICs
Think long‐term, be flexible and plan for continuity	1) Long‐term investment either by in‐country funders or external funders is necessary; 2) computational modelling career track pipeline allows for sustainability; 3) involve in‐country academic institutions	Short term investments, such as workshops and short course trainings not affiliated with academic institutions in‐country do not allow for sustainable capacity building

HIC, High Income Country; LMICs, Low‐ and middle‐income countries; MOH, Ministry of Health.

a The seven principles for strengthening research capacity in low‐ and middle‐income countries: simple ideas in a complex world (2014) by ESSENCE on Health Research is licensed by the Wellcome Trust of the United Kingdom under a Creative Commons Attribution‐NonCommercial‐ShareAlike 3.0 Unported License. (http://www.who.int/tdr/partnerships/initiatives/essence/en/);

b these data were collected from CRDF Global (OISE‐17‐62962‐1, OISE‐17‐62965‐1, OISE‐17‐62967‐1) grantees through progress reports, a short survey and follow‐up interviews.

Fogarty International Center has also led modelling efforts like the Multinational Influenza Seasonal Mortality Study and the Research and Policy in Infectious Disease Dynamics programme, which have generated research activities and training workshops to build a network of global experts in infectious disease modelling [14]. Establishing a similar modelling community in LMICs focused on integrated HIV care is only starting, and, from our experiences with 136 workshops [14] we have summarized the following recommendations to strengthen and sustain progress.

1. Modellers should make modelling tools and analytic packages publicly available to wider audiences. Accessible user‐friendly tools should be developed that address NCD care for PLHIV in LMICs, ideally by modellers in LMICs once capacitated. This could be achieved by modifying generic policy modelling tools such as the Spectrum software to incorporate more specific questions of interest to this region. Furthermore, existing simulation tools should be repackaged into workflows with convenient user interface, visualization components and reporting modules.
2. Like the experiences of the grantees above working on HIV/NCD integrated care, working on infectious disease modelling we have found that local training programmes should be developed at LMIC academic centres affiliated with universities to grow the next cohort of LMIC modellers [13, 14, 15]. These programmes should include master’s and Ph.D. programmes in computational modelling [15]. A notable example is the South African Center for Excellence in Epidemiological Modelling and Analysis (SACEMA), a multidisciplinary research centre hosted at Stellenbosch University, focused on modelling interventions to improve health in Africa. SACEMA offers “modelling clinics” for African participants who bring a dataset and research questions for analysis in Stellenbosch, South Africa, and are encouraged to continue their modelling efforts at their home institutions [16]. SACEMA also hosts several research fellows in‐residence [16].
3. We found, in our experience, that 1.5 days to 1 week‐long trainings in disease modelling along with student exchanges with expert groups in HICs, are beneficial to begin to grow modelling in LMICs. However, they cannot replace formal academic training locally [14]. Although these trainings are rather short to provide extensive instruction in disease modelling, it is seen as a reasonable amount of time that students and professionals can take away from their regular duties. Short workshops can also help identify participants who are particularly interested in the field of computational modelling and can be offered further trainings and visits to HIC institutions to deepen their expertise.
4. Training of decision makers to understand model outputs, particularly uncertainty and confidence intervals, will allow them to be confident when using model estimates to make decisions and help them to trust modelling.

Computational modelling is a relatively fast, low‐cost solution to answer questions about HIV/NCD integrated care, which are mentioned above and can also provide future projections useful for planning. Researchers in‐country must be part of the process of developing and using models, ultimately in a leadership role, requiring training efforts to empower researchers and strengthen institutional capacity. Policy makers need to be educated about the advantages and limitations of modelling estimates to allow them to use model outputs appropriately for decision‐making. Finally, collection of new datasets on NCDs in PLHIV in LMICs will be essential as models are only as good as the underlying data they rely on. Now is the time to invest in data integration and capacity building for mathematical modelling in LMICs to enable more science‐based decisions for holistic treatment of PLHIV and the entire population.

## Competing interests

None of the authors have competing interests to declare.

## Authors’ Contributions

BB, CV, PB, XW and LK conceived, wrote, reviewed, read and approved the manuscript. All contributed equally. All authors have read and approved the final manuscript.

## Abbreviations

PLHIV, People living with HIV; NCD, Non‐communicable diseases; LMICs‐ Low‐ and middle‐income countries; NIH‐ US National Institutes of Health; FIC, Fogarty International Center; HIC, High‐income country; SACEMA, South African Center for Excellence in Epidemiological Modelling and Analysis.

## Funding

The authors have not declared a specific grant for this research from any funding agency in the public, commercial or not‐for‐profit sectors.

## Disclaimer

The findings and conclusions in this manuscript are those of the author(s) and do not necessarily represent the official position or policy of the US National Institutes of Health, the US Department of Health and Human Services or any other institutions with which the authors are affiliated.
